# Reviewers BJCVS 32.3

**Published:** 2017

The Brazilian Journal of Cardiovascular Surgery (BJCVS) is grateful for the reviewers, listed below, which collaborated in this edition. Without their work would be impossible to keep the high scientific standard of our journal.

Carla Tanamati
Claudio Gelape
Diego Gaia
Edmo Gabriel
João de Deus e Brito
Leonardo Miana
Luís Dallan
Luiz Felipe Moreira
Melchior Lima
Michel Pompeu B. de O. Sá
Paulo Roberto Evora
Robison Poffo
Valquíria Campagnucci

